# The MAPP research network: a novel study of urologic chronic pelvic pain syndromes

**DOI:** 10.1186/1471-2490-14-57

**Published:** 2014-08-01

**Authors:** J Quentin Clemens, Chris Mullins, John W Kusek, Ziya Kirkali, Emeran A Mayer, Larissa V Rodríguez, David J Klumpp, Anthony J Schaeffer, Karl J Kreder, Dedra Buchwald, Gerald L Andriole, M Scott Lucia, J Richard Landis, Daniel J Clauw

**Affiliations:** 1 Department of Urology, University of Michigan, Ann Arbor, MI, USA; 2 National Institute of Diabetes and Digestive and Kidney Diseases, National Institutes of Health, Bethesda, MD, USA; 3 Division of Digestive Diseases, University of California, Los Angeles, CA, USA; 4 Department of Urology, Northwestern University, Chicago, IL, USA; 5 Department of Urology, University of Iowa, Iowa City, IA, USA; 6 Departments of Epidemiology and Medicine, University of Washington, Seattle, WA, USA; 7 Division of Urologic Surgery, Department of Surgery, Washington University School of Medicine, St Louis, MO, USA; 8 Department of Pathology, University of Colorado Anschutz Medical Campus, Aurora, CO, USA; 9 Department of Biostatistics and Epidemiology, University of Pennsylvania Perelman School of Medicine, Philadelphia, PA, USA; 10 Departments of Anesthesiology and Medicine, University of Michigan, Ann Arbor, MI, USA

**Keywords:** Urological chronic pelvic pain syndromes, Interstitial cystitis, Chronic prostatitis, Translational research, Multi-disciplinary

## Abstract

Urologic chronic pelvic pain syndrome (UCPPS) may be defined to include interstitial cystitis/bladder pain syndrome (IC/BPS) and chronic prostatitis/chronic pelvic pain syndrome (CP/CPPS). The hallmark symptom of UCPPS is chronic pain in the pelvis, urogenital floor, or external genitalia often accompanied by lower urinary tract symptoms. Despite numerous past basic and clinical research studies there is no broadly identifiable organ-specific pathology or understanding of etiology or risk factors for UCPPS, and diagnosis relies primarily on patient reported symptoms. In addition, there are no generally effective therapies. Recent findings have, however, revealed associations between UCPPS and “centralized” chronic pain disorders, suggesting UCPPS may represent a local manifestation of more widespread pathology in some patients. Here, we describe a new and novel effort initiated by the National Institute of Diabetes and Digestive and Kidney Diseases (NIDDK) of the U.S. National Institutes of Health (NIH) to address the many long standing questions regarding UCPPS, the Multidisciplinary Approach to the Study of Chronic Pelvic Pain (MAPP) Research Network. The MAPP Network approaches UCPPS in a systemic manner, in which the interplay between the genitourinary system and other physiological systems is emphasized. The network’s study design expands beyond previous research, which has primarily focused on urologic organs and tissues, to utilize integrated approaches to define patient phenotypes, identify clinically-relevant subgroups, and better understand treated natural history and pathophysiology. Thus, the MAPP Network provides an unprecedented, multi-layered characterization of UCPPS. Knowledge gained is expected to provide important insights into underlying pathophysiology, a foundation for better segmenting patients for future clinical trials, and ultimately translation into improved clinical management. In addition, the MAPP Network’s integrated multi-disciplinary research approach may serve as a model for studies of urologic and non-urologic disorders that have proven refractory to past basic and clinical study.

**Trial registration:**

ClinicalTrials.gov identifier: NCT01098279 “Chronic Pelvic Pain Study of Individuals with Diagnoses or Symptoms of Interstitial Cystitis and/or Chronic Prostatitis (MAPP-EP)”.

## Background

Urologic chronic pelvic pain syndrome (UCPPS) encompasses two highly prevalent non-malignant urologic disorders, interstitial cystitis/bladder pain syndrome (IC/BPS) and chronic prostatitis/chronic pelvic pain syndrome (CP/CPPS). UCPPS is primarily characterized by chronic and often debilitating pain in the pelvic region and/or genitalia and typically a spectrum of defects in bladder and lower urinary tract function [1,2].

Numerous studies have been conducted over the past two decades to define the pathophysiology and natural history of UCPPS and to examine the efficacy of therapies. Many of those studies were supported by the National Institute of Diabetes and Digestive and Kidney Diseases (NIDDK) of the U.S. National Institutes of Health (NIH). The first NIDDK-sponsored pelvic pain clinical research network, the Interstitial Cystitis Database study (ICDB) was initiated in 1991 [3]. This five-year prospective cohort study collected data on more than 600 persons and characterized them across demographic and clinical characteristics, including bladder biopsy [4]. The Interstitial Cystitis Clinical Trials Group (ICCTG) was subsequently established to conduct randomized clinical trials beginning in 1996 [5-9]. In 2003, this group became the Interstitial Cystitis Collaborative Research Network (ICCRN) and carried out additional randomized clinical trials [10-12]. In 1998, the Chronic Prostatitis Cohort (CPC) study began to prospectively collect patient data to systematically examine the demographics, clinical characteristics and natural history of CP/CPPS [13]. The NIDDK subsequently initiated the Chronic Prostatitis Collaborative Research Network (CPCRN) which performed clinical trials for CP/CPPS [14-17]. Results from these clinical research studies failed to identify definitive risk factors or generally effective treatments, with the exception of a single study suggesting that myofascial physical therapy might be effective in IC/BPS [18]. The NIDDK-supported Boston Area Community Health (BACH) Survey [19-21] and the RAND IC Epidemiology (RICE) Study [22,23] provided estimates on the prevalence of IC-related symptoms for both men and women, as well as an expanded understanding of symptom morbidity. In addition to these clinical and epidemiological studies, many basic research efforts were developed to describe pathophysiology at the cellular level, including *in vivo* studies of model systems. However, no consensus agreement has been achieved on an underlying etiology for UCPPS, though co-occurrence of UCPPS with other chronic non-urologic pain syndromes has been revealed [24-30].

In light of the limitations of previous studies and results showing potential associations between UCPPS and other chronic pain conditions, the NIDDK proposes that the traditional bladder and prostate centered focus of UCPPS research be broadened to a systemic view of disease in which the interplay between the genitourinary system and other physiological systems (e.g., the central nervous system), is highlighted. In addition, it is suggested that studies of UCPPS would benefit from incorporating broad approaches involving a diversity of urologic and non-urologic disciplines to promote a more comprehensive characterization of patient phenotype.

These concepts, as well as recommendations solicited from the scientific community [31], prompted the NIDDK to initiate a new research program for the study of UCPPS, the Multidisciplinary Approach to the Study of Chronic Pelvic Pain (MAPP) Research Network. Since its inception in 2008, the MAPP Research Network has adopted a highly collaborative and integrated research strategy that incorporates new and novel approaches conducted by investigators representing traditional urologic disciplines and broad non-urologic expertise, including experts in pain research, neurobiology and neuroimaging, infectious disease, biomarker discovery, animal modeling, epidemiology, psychology, immunology, among many others. The overarching goal of the MAPP Research Network is to provide findings useful for designing future clinical trials and ultimately to improve clinical management for UCPPS patients. Importantly, the design and goals of the MAPP Network are complementary to other large phenotyping efforts for non-urologic pain conditions being conducted, such as the OPPERA study [32].

The MAPP Research Network includes six Discovery Sites and several specialized sub-sites that conduct multiple, collaborative Trans-MAPP (i.e., across sites) studies, as well as a number of single-site studies, and two specialized Cores (see Acknowledgement for complete listing of MAPP Network Sites and affiliated personnel). The Data Coordination Core (DCC) serves as the central site for data acquisition and storage; provides bio-statistical analyses for all studies; and promotes network-wide quality assurance. The DCC also provides administrative support, including development and maintenance of a public website (http://www.mappnetwork.org/). The Tissue Analysis and Technology Core (TATC) monitors biosample collection and provides sample banking, annotation, and distribution services.

The MAPP Research Network is currently conducting complementary basic, translational, and clinical science studies to investigate questions of significant clinical relevance and adopts the view that UCPPS potentially involves significant systemic contributions. The primary scientific protocol is a prospective observational study of the treated natural history UCPPS, the Trans-MAPP Epidemiology/Phenotyping (EP) Study. A full description of the central Trans-MAPP EP Study and the complement of urologic and non-urologic measures employed are described in the companion report by Landis et al. [33]. In addition to the extensive phenotyping, the Trans-MAPP EP Study also provide a source of highly characterized participants for further phenotyping through other integrated network protocols. Assembled network working groups develop and conduct complementary Trans-MAPP studies that broadly address potential contributions of various physiological systems and hypotheses of underlying etiology, pathophysiology, risk, and relationships between UCPPS and commonly associated non-urologic syndromes. These include structural and functional assessments of the central nervous system; efforts to uncover potential contributions of infectious agents to etiology; discovery efforts to identify new biological markers; extensive characterizations of symptom variation (e.g., flares); and efforts to develop new and more informative patient reported outcome measures. The network is also engaged in collaborative research to establish and assess animal models validated for the presence of clinically-relevant phenotypes, thus allowing for improved translation between animal and human studies. The MAPP study will provide comprehensive, state-of-the-art phenotyping data which will set the standard for future UCPPS research. Results from the MAPP study can be integrated with other, more clinically focused phenotyping efforts (such as the UPOINT system [34,35]) to better define the ‘minimal data set’ required to provide optimal patient care for UCPPS patients.

Within the MAPP Research Network all clinical Trans-MAPP protocols and site-specific efforts, which primarily serve to pilot test ideas complementary to the collaborative protocols, are highly integrated through their use of shared patients and controls evaluated through standard phenotyping; common biological samples; and a standardized data collection, storage, and analysis strategy. In addition, neuroimaging study parameters are standardized across sites and scan data is centrally managed by the University of California at Los Angeles (UCLA) Center for Neurobiology of Stress (painrepository.org), in close collaboration with UCLA-Laboratory of Neuroimaging (LONI), which has extensive experience in the collection, storage and analysis of large multi-site MRI data sets (loni.usc.edu). In this way diverse findings across protocols may be integrated to allow a detailed characterization of a single UCPPS patient or patient sub-groups. Importantly, these efforts are also generating a unique national resource of highly detailed longitudinal clinical and epidemiological data associated with data from additional, integrated phenotyping studies and linked biological samples, for future use by the wider research community through the NIDDK Data and Sample Repositories (http://www3.niddk.nih.gov/researchprograms/repositories/).

## Conclusions

UCPPS research is clearly at a cross-road in which the traditional basic and clinical scientific strategies are being re-evaluated in light of evolving ideas of UCPPS and recognition of the limitations of previous study designs. The MAPP Research Network was created to address these challenges. Advances from network efforts are expected to provide a more comprehensive understanding of UCPPS pathophysiology, identify clinically relevant patient sub-groups, inform the design of future clinical trials, and ultimately improve clinical care. The unique organization and approach of the MAPP Network may also provide a blueprint for multi-site, multi-disciplinary research in the broader pain field, as well as for those disciplines addressing other disorders with ill-defined pathophysiology.

## Appendix: MAPP Research Network Study Group

### MAPP Network Executive Committee

J. Quentin Clemens, MD, FACS, MSci,

Network Chair, 2013-Philip Hanno, MD

Ziya Kirkali, MD

John W. Kusek, PhD

J. Richard Landis, PhD

M. Scott Lucia, MD

Chris Mullins, PhD

Michel A. Pontari, MD

### Northwestern University Discovery Site

David J. Klumpp, PhD, Co-Director

Anthony J. Schaeffer, MD, Co-Director

Apkar (Vania) Apkarian, PhD

David Cella, PhD

Melissa A. Farmer, PhD

Colleen Fitzgerald, MD

Richard Gershon, PhD

James W. Griffith, PhD

Charles J. Heckman II, PhD

Mingchen Jiang, PhD

Laurie Keefer, PhD

Darlene S. Marko, RN, BSN, CCRC

Jean Michniewicz

Todd Parrish, PhD

Frank Tu, MD, MPH

### University of California, Los Angeles Discovery Site and PAIN Neuroimaging Core

Emeran A. Mayer, MD, Co-Director

Larissa V. Rodríguez, MD, Co-Director

Jeffry Alger, PhD

Cody P. Ashe-McNalley

Ben Ellingson, PhD

Nuwanthi Heendeniya

Lisa Kilpatrick, PhD

Jason Kutch, PhD

Jennifer S. Labus, PhD

Bruce D. Naliboff, PhD

Fornessa Randal

Suzanne R. Smith, RN, NP

### University of Iowa Discovery Site

Karl J. Kreder, MD, MBA, Director

Catherine S. Bradley, MD, MSCE

Mary Eno, RN, RA II

Kris Greiner, BA

Yi Luo, PhD, MD

Susan K. Lutgendorf, PhD

Michael A. O’Donnell, MD

Barbara Ziegler, BA

### University of Michigan Discovery Site

Daniel J. Clauw, MD, Co-Director;

Network Chair, 2008-2013

J. Quentin Clemens, MD, FACS, MSci,

Co-Director; Network Chair, 2013-

Suzie As-Sanie, MD

Sandra Berry, MA

Megan E. Halvorson, BS, CCRP

Richard Harris, PhD

Steve Harte, PhD

Eric Ichesco, BS

Ann Oldendorf, MD

Katherine A. Scott, RN, BSN

David A. Williams, PhD

### University of Washington, Seattle Discovery Site

Dedra Buchwald, MD, Director

Niloofar Afari, PhD, Univ. Of California, San Diego

John Krieger, MD

Jane Miller, MD

Stephanie Richey, BS

Susan O. Ross, RN, MN

Roberta Spiro, MS

TJ Sundsvold, MPH

Eric Strachan, PhD

Claire C. Yang, MD

### Washington University, St. Louis Discovery Site

Gerald L. Andriole, MD, Co-Director

H. Henry Lai, MD, Co-Director

Rebecca L. Bristol, BA, BS, Coordinator

Graham Colditz, MD, DrPH

Georg Deutsch, PhD, Univ. of Alabama at Birmingham

Vivien C. Gardner, RN, BSN, Coordinator

Robert W. Gereau IV, PhD

Jeffrey P Henderson, MD, PhD

Barry A. Hong, PhD, FAACP

Thomas M. Hooton, MD, Univ of Miami

Timothy J. Ness, MD, PhD, Univ. of Alabama at Birmingham

Carol S. North, MD, MPE, Univ. Texas Southwestern

Theresa M. Spitznagle, PT, DPT, WCS

Siobhan Sutcliffe, PhD, ScM, MHS

### University of Pennsylvania Data Coordinating Core (DCC)

J. Richard Landis, PhD, Core Director

Ted Barrell, BA

Philip Hanno, MD

Xiaoling Hou, MS

Tamara Howard, MPH

Michel A. Pontari, MD

Nancy Robinson, PhD

Alisa Stephens, PhD

Yanli Wang, MS

### University of Colorado Denver Tissue Analysis & Technology Core (TATC)

M. Scott Lucia, MD, Core Director

Adrie van Bokhoven, PhD

Andrea A. Osypuk, BS

Robert Dayton, Jr

Karen R. Jonscher, PhD

Holly T. Sullivan, BS

R. Storey Wilson, MS

### Additional Sites: Drexel University College of Medicine

Garth D.Ehrlich, PhD

### Harvard Medical School/Boston Children’s Hospital

Marsha A. Moses, PhD, Director

Andrew C. Briscoe

David Briscoe, MD

Adam Curatolo, BA

John Froehlich, PhD

Richard S. Lee, MD

Monisha Sachdev, BS

Keith R. Solomon, PhD

Hanno Steen, PhD

### Stanford University

Sean Mackey, MD, PhD, Director

Epifanio Bagarinao, PhD

Lauren C. Foster, BA

Emily Hubbard, BA

Kevin A. Johnson, PhD, RN

Katherine T. Martucci, PhD

Rebecca L. McCue, BA

Rachel R. Moericke, MA

Aneesha Nilakantan, BA

Noorulain Noor, BS

### Queens University

J. Curtis Nickel, MD, FRCSC, Director

### National Institutes of Diabetes and Digestive and Kidney Diseases (NIDDK), National Institutes of Health (NIH)

Chris Mullins, PhD

John W. Kusek, PhD

Ziya Kirkali, MD

Tamara G. Bavendam, MD

## Abbreviations

BACH: Boston area community health survey; BPS: Bladder pain syndrome; CFS: Chronic fatigue syndrome; CNS: Central nervous system; CPC: Chronic prostatitis cohort; CP/CPPS: Chronic prostatitis/chronic pelvic pain syndrome; DCC: Data coordinating core; DNA: Deoxyribonucleic acid; EEP: External experts panel; FM: Fibromyalgia; IBS: Irritable bowel syndrome; IC/BPS: Interstitial cystitis/bladder pain syndrome; ICCRN: Interstitial cystitis collaborative research network; ICCTG: The interstitial cystitis clinical trials group; ICDB: Interstitial cystitis database study; LONI: Laboratory of neuroimaging; MAPP: Multidisciplinary approach to the study of chronic pelvic pain; MRI/fMRI: Magnetic resonance imaging/functional magnetic resonance imaging; NIDDK: The national institute of diabetes, digestive, and kidney diseases; NUAS: Non-urologic associated syndromes; PPT: Pressure pain threshold; RICE: RAND IC epidemiology study; TATC: Tissue analysis and technology core; Trans-MAPP EP: Trans-MAPP epidemiology and phenotyping study; UCLA: University of california at Los Angeles; UCPPS: Urological chronic pelvic pain syndromes; VB1, VB2: Voided bladder 1, 2

## Competing interests

JQ Clemens, C Mullins, JW Kusek, Z Kirkali, EA Mayer, LV Rodriguez, AJ Schaeffer, D Buchwald, and JR Landis declare no competing interests. DJ Klumpp declares ownership and equity interests in ProbioTx Inc, and Gold Coast Therapeutics Inc. KJ Kreder is a Consultant for Medtronic, Astellas, Symptelligence, and Tengion. GL Andriole is a Consultant for Augmenix, Bayer, Genomic Health, GlaxoSmithKline and Myriad Genetics and has received research grants from Johnson & Johnson, Medivation and Wilex. MS Lucia declares ownership of 3D Biopsy and has consulted for Myriad Genetics and Bayer Healthcare. DJ Clauw has received grants from Pfizer, Cerephex, Lilly, Merck, Nuvo and Furest, and Consulting Fees and Honoraria from Pfizer, Cerephex, Lilly, Merck, Nuvo, Furest, Tonix, Purdue, Therauance, and Johnson & Johnson.

## Authors’ contributions

JQC wrote the initial draft manuscript. All authors read and approved the final manuscript.

## Pre-publication history

The pre-publication history for this paper can be accessed here:

http://www.biomedcentral.com/1471-2490/14/57/prepub
